# Sepsis prevents the development of experimental type 1 diabetes

**DOI:** 10.3389/fimmu.2025.1658960

**Published:** 2025-10-09

**Authors:** Goran Stegnjaić, Dragica Mićanović, Tamara Saksida, Sanja Despotović, Thomas S. Griffith, Vladimir P. Badovinac, Đorđe Miljković, Suzana Stanisavljević

**Affiliations:** ^1^ Department of Immunology, Institute for Biological Research “Siniša Stanković” - National Institute of Republic of Serbia, University of Belgrade, Belgrade, Serbia; ^2^ Institute of Histology and Embryology “Aleksandar Đ. Kostić”, Faculty of Medicine, University of Belgrade, Belgrade, Serbia; ^3^ Department of Urology, Center for Immunology, University of Minnesota, Minneapolis, MN, United States; ^4^ Department of Pathology, University of Iowa, Iowa City, IA, United States

**Keywords:** type 1 diabetes, sepsis, T cells, autoimmunity, cecal ligation and puncture

## Abstract

**Introduction:**

While both sepsis and autoimmunity are characterized by dysregulated immune responses, their mutual influence remains only partially understood.

**Methods:**

In this study, we investigated how sepsis affects the development of type 1 diabetes (T1D), an autoimmune disease characterized by the destruction of pancreatic β-cells. Specifically, we examined the impact of polymicrobial sepsis on the progression of T1D induced by multiple low doses of streptozotocin (MLDS). C57BL/6 mice were subjected to cecal ligation and puncture (CLP) to induce sepsis, and T1D was subsequently induced using the MLDS protocol after the mice had fully recovered from the acute phase of sepsis.

**Results and discussion:**

Although CLP triggered transient hypoglycemia, it did not impair the structure or function of the endocrine pancreas, and the mice were normoglycemic at the time of T1D induction. Notably, CLP limited immune cell infiltration into the pancreas following MLDS treatment, thereby preventing the onset of T1D and the development of hyperglycemia. CD4^+^ T cells are important for initiating the autoimmune attack on pancreatic islets by activating CD8^+^ T cells and macrophages. Thus, it seems plausible that the protective effect observed in CLP-exposed mice was due to the reduction in CD4^+^ T cells, but not CD8^+^ T cells, in the pancreatic lymph nodes. Additionally, CD4^+^ T cells and regulatory T cells in the spleens of CLP-treated mice exhibited elevated expression of inhibitory/exhaustion markers. These findings suggest that sepsis-induced alterations in the CD4^+^ T cell compartment within pancreas-associated lymphoid organs confer protection against MLDS-induced T1D.

## 1 Introduction

Type 1 diabetes (T1D) is an autoimmune disease characterized by the destruction of pancreatic β-cells and disruption of insulin-regulated blood glucose levels. T1D usually affects children and young adults, but it can also develop in people of older age (2). T1D accounts for ~5-10% of total diabetic cases worldwide, although diverse distributions can be observed in different countries (3). The exact cause of T1D is not yet fully understood, but the combination of genetic and environmental factors, such as viral infections or other triggers, have been suggested to initiate the autoimmune response against the pancreatic β-cells (4). This response leads to the infiltration of immune cells, including autoreactive T and B cells and macrophages, which attack and destroy pancreatic β-cells (4–7). The β-cells are gradually destroyed over time, leading to insufficient insulin production which impairs glucose regulation. Despite continuing effort to develop novel therapies for T1D, such as monoclonal antibodies or vaccines (8), the only therapy available for the vast majority of T1D patients is insulin supplementation for the life of the individual. Therefore, there is a continuing need to uncover the pathological mechanisms of the autoimmune response and find new therapeutic approaches for T1D.

Multiple immunotherapies have been proposed, such as antibody-based (an anti-CTLA-4 antibody - Abatacept, anti-CD3 monoclonal antibodies – Teplizumab and Otelixizumab, anti-CD20 antibody - Rituximab), those acting against pro-inflammatory cytokines (IL-1β inhibitors - Anakinra and Canakinumab, TNF blockers - Etanercept and Infliximab), regulatory T cells (Treg)-mediated (e.g. Chimeric Antigen Receptor (CAR)-Treg therapy), antigen-based therapies (GAD-based vaccines, BCG vaccine, autoantigen treatments), islet transplantation and stem cell therapies, yet with limited efficiency (9, 10). The common goal of these therapeutic approaches is to restore adaptive immune balance, predominantly through promoting Treg activity and reducing actions of effector and effector memory T cells (11).

Sepsis is characterized by a cytokine storm that precedes lymphopenia and immune cell functional deficits. This state of immunoparalysis is prolonged and affects the ability of the immune system to respond to infections and cancer (12). Although sepsis is a life-threatening condition, there are indications that the sepsis-induced changes in cellular immune response may be beneficial in regulating autoimmune responses. However, the relationship between sepsis and autoimmune diseases is not completely understood (13). Some epidemiological studies are showing worse clinical outcomes of sepsis in patients with autoimmune diseases, while in some studies there are no differences in septic outcome in patients with autoimmune diseases compared to healthy individuals (14–16). Data about the influence of previous septic events on autoimmune disease is even sparser, making this connection elusive. Recently we have shown that an autoimmune response can be altered by prior exposure of animals to sepsis (17). Specifically, animals were exposed to polymicrobial sepsis induced by cecal ligation and puncture (CLP) before the induction of experimental autoimmune encephalomyelitis (EAE), an animal model of central nervous system autoimmunity. These animals had reduced numbers of naïve autoantigen-specific T cells, which resulted in impairment of the T cell response to autoantigen and amelioration of EAE. There are several studies showing that T cell exhaustion could be harnessed for treatment of T1D (18). These studies focused on the exhausted phenotype of CD8^+^ T cells since they mediate β-cell killing in pancreas. However, there is a dearth of knowledge whether exhaustion of CD4^+^ T cells could ameliorate autoimmune response in T1D. It is well known that CD4^+^ T cells are implicated in T1D because of the strong association of T1D with HLA class II, and of their role in providing “help” to CD8^+^ T cells and B cells. This is why it could be important to observe if the CD4^+^ T cell exhaustion has beneficial effects in T1D.

Having in mind that CLP was able to restrain autoimmune response in EAE, we aimed to examine the effects of septic immunoparalysis on the pathogenic processes in another autoimmune disease, T1D. For this purpose, we used multiple low-dose streptozotocin (MLDS) model of T1D in C57BL/6 mice. In this model, as well as in T1D in humans, it is suggested that CD4^+^ T cells specific for β-cell autoantigens have pro-inflammatory phenotype characterized by the secretion of IFN-γ and/or IL-17 (1, 19–21). Thus, besides investigating the influence of sepsis on the development of hyperglycemia and insulitis, we focused on the analysis of immune cells, particularly T cells, in the pancreatic lymph nodes (PLN) and spleen.

## 2 Material and methods

### 2.1 Experimental animals and CLP

C57BL/6 mice were from the animal facility of the Institute for Biological Research “Sinisa Stankovic” - National Institute of the Republic of Serbia, University of Belgrade. All experiments were approved by the Veterinary Administration of the Ministry of Agriculture, Forestry and Water Management, Republic of Serbia (No 323-07-02150/2023-05).

Polymicrobial sepsis was induced in 8–12-week-old male mice by CLP (22). Mice were anesthetized by isoflurane inhalation (1000 mg/g Vetflurane, Virbac, France). The abdomen was shaved and disinfected with Iodine (10% Povidon jod HF, Hemofarm AD, Vršac, Serbia), and a ~1cm midline incision was made. The distal third of the cecum was ligated with a 4–0 PGLA lactic suture (DKO241PL, Yavo, Poland). The cecum was punctured with a 26G needle (Sinomedic, Sinofarm, Belgrade, Serbia), and a small amount of the cecal content, the size of a droplet, was extruded. The cecum was returned to the abdomen, and the peritoneum was closed with a 4–0 PGLA lactic uninterrupted suture (DKO241PL, Yavo, Poland). The skin was sealed using tissue adhesive (3M Vetbond, Japan). At the site of the incision 20 µl of local anesthetic (0.5% lidocaine, Galenika, Belgrade, Serbia) was administered. This procedure creates a septic state characterized by loss of appetite and body weight, ruffled fur, shivering, diarrhea, and/or periorbital exudates with up to 20% mortality rate (15). The low mortality rate was purposely aimed in order to obtain an adequate number of animals for the induction of T1D, with as little as possible used in the experiments. Although mortality was relatively low, notable sepsis signs, as defined below, were evident in all of CLP-treated mice. Sham mice underwent identical surgery, excluding cecal ligation and puncture.

Clinical signs of sepsis in the mice were monitored daily, from the day of the CLP surgery until day 8 post surgery. For the evaluation of sepsis, 4 distinct parameters were scored as follows: grooming - 0, normal fur; 1, “dusty”, fur that has lost shine and looks matte; 2, “ruffled”, fur that becomes erect; mobility – 0, normal; 1, reduced mobility; 2, mice are immobile; body position – 0, normal; 1, back is arched; 2, an animal is laying on the side; weight loss – 0, <10%; 1, 10-15%; 2, >15%. After giving one score for each category, the sum of all categories indicates the disease score. Importantly, dead mice are given the highest score (8) on the day of death and after that are removed from scoring. Healthy scores range from 0 to 2, moderate disease scores range from 3 to 5, and severe disease scores range from 6 to 8. Along with clinical signs of sepsis, blood glucose from tail vein was measured in the same time period.

### 2.2 MLDS-induced T1D

T1D was induced by injecting streptozotocin (STZ, 38 mg/kg of body weight intraperitoneally) five consecutive days, as previously described (23). STZ treatment started on day 12 after the CLP procedure, at the time when mice showed no clinical signs of sepsis. STZ (Sigma-Aldrich, St. Louis, MO, USA) was dissolved in a cold 0.1 M citrate phosphate solution (pH 6) right before the treatment. Mice were monitored for their body mass and blood glucose level once a week. Blood from the tail vein was used for glycaemia assessment by GlucoSure AutoCode glucometer (ApexBio, Hsinchu City, Taiwan).

### 2.3 Glucose tolerance test

Tolerance to glucose was performed on fasted mice by intraperitoneal injection of D-glucose (2 mg/g bw, Sigma–Aldrich, St. Louis, MO). Blood glucose from the tail vein was measured before glucose administration and at specific time points after the administration (15, 30, 60 and 120 minutes).

### 2.4 Histology

Pancreata were aseptically removed on days 1, 3 and 12 post-CLP, and on day 42 after the induction of T1D. The tissue was fixed in neutral buffered formalin and then embedded in paraffin, sectioned (5 µm thick), and stained with Hematoxylin and Eosin (H&E; Bio-Optica, Milano, Italy) or immunohistochemically with anti-insulin and anti-glucagon antibodies. Sections were photographed using a Leica DM4000 B LED light microscope (Leica, Wetzlar, Germany), and a Leica DFC295 digital camera (Leica, Heerbrugg, Switzerland).

The incidence and degree of inflammatory changes in pancreatic islets were assessed on H&E-stained slides. Insulitis scoring was performed by examining fifteen islets per mouse and graded in a blinded fashion as follows: 0- no infiltrate (healthy islets), 1- periductular infiltrate, 2- peri-islet infiltrate (peri-insulitis). Results are presented as a percentage of graded islets per total number of islets, with three pancreata examined per group.

Immunohistochemical staining for insulin and glucagon was performed on adjacent sections. Briefly, slides were deparaffinised and rehydrated; heat-induced antigen retrieval was done in a microwave in 0.01 M sodium citrate buffer (pH 6.0) for 20 minutes at 750W. Endogenous peroxidase was blocked with commercial blocker (Envision FLEX, Dako, Glostrup, Denmark). Slides were then incubated overnight with primary antibodies (anti-insulin (H-86) antibody 1:500; anti-glucagon (FL-180) antibody 1:500; both from Santa Cruz, CA), followed by incubation with peroxidase-conjugated secondary antibodies (Envision FLEX HRP, Dako) according to the manufacturer’s instructions. Staining was developed with diaminobenzidine (Envision FLEX, Dako). Sections were counterstained with Meyer’s hematoxylin (BioOptica, Milano, Italy). On immunohistochemically stained sections, the number of insulin and glucagon cells was counted on 40 sections from 4 mice per group, and expressed as the percentage of insulin/glucagon-positive cells per total number of cells in the islet.

### 2.5 Isolation of cells and cell cultures

Pancreatic lymph node cells (PLNC) and splenocytes (SPC) were isolated from mice 9 days after the first injection of streptozotocin. Lymph nodes and splenic tissues were dispersed through a plastic cell strainer (70 µm). Erythrocytes from the SPC pellet were removed by Ammonium-Chloride-Potassium (ACK) lysis buffer (0.15M Ammonium chloride, 0.01M Potassium bicarbonate, 0.1mM Disodium EDTA). Cell suspensions were centrifuged at 550g for 5 minutes. The absolute number of live PLNC and SPC was determined by trypan blue exclusion counting in a Bürker-Türk counting chamber.

SPC were incubated in RPMI-1640 medium (Capricorn Scientific, Ebsdorfergrund, Germany) supplemented with 5% fetal bovine serum, in 24-well plates (5x10^6^/ml, Sarstedt, Pasching, Austria), and stimulated with Concanavalin A (1 μg/mL, Sigma-Aldrich). Cell culture supernatants were obtained 24 hours later.

Cells were treated for 4 hours for intracellular cytokine detection with a 500x diluted eBioscience™ Cell Stimulation Cocktail containing phorbol 12-myristate 13-acetate (PMA), ionomycin, and a protein transport inhibitor (Thermo Fisher Scientific, Waltham, MA). All incubations were performed at 37°C in a humidified atmosphere (5% CO2).

### 2.6 ELISA

SPC cell-free culture supernatants were obtained by centrifugation at 550 g for 3 minutes. Cytokine concentration in the supernatant was determined by ELISA using MaxiSorp plates (Nunc, Rochild, Denmark) and appropriate capture and detection antibodies for interferon (IFN)-γ, and interleukin (IL)-17 (Thermo Fisher Scientific). Blood was collected retro-orbitally, left to clot for 20 minutes at room temperature, and then centrifuged at 1500 g for 10 minutes, at 4˚C. Serum was collected and stored frozen until used. Appropriate capture and detection antibodies for IL-1β (Biolegend, San Diego, CA), IL-6, and TNF (R&D Systems, Minneapolis, MN) were used according to the manufacturer’s instructions. Standard curves were generated with known concentrations of recombinant cytokines. The absorbance was measured at 450 nm with a correction at 670 nm using a Synergy H1 multi-plate reader H1 (Agilent Technologies, Santa Clara, CA). Samples were analyzed in duplicate. The lower and upper detection limits were 30 pg/mL and 10 ng/mL, respectively.

### 2.7 Cytofluorimetry

PLNC and SPC were stained with the antibodies listed in **Table 1** according to the procedure suggested by the manufacturers. The incubation for surface staining lasted 30 minutes at 4°C. The incubation for intracellular staining lasted 40 minutes at 4°C. Intracellular staining for cytokines and FoxP3 was performed with eBioscience™ Foxp3/Transcription Factor Fixation/Permeabilization Concentrate and Diluent, eBioscience™ Intracellular Fixation & Permeabilization Buffer Set, and eBioscience™ Permeabilization Buffer (10X) (all from Thermo Fisher Scientific), according to manufacturer recommendations. Appropriate isotype control antibodies were used as required to establish gates for cell marker positivity. Sample acquisition was performed using a CytoFLEX flow cytometer (Beckman Coulter, Indianapolis, IN) and analyzed using CytExpert software (Beckman Coulter). Cytofluorimetry results are presented as the number of cells bound by an appropriate antibody. The number of cells in a specific cell population was calculated using the absolute number of PLNC or SPC and the percentage of the specific population within live singlet cells obtained by flow cytometry. Representative plots for gating strategies are provided in the **Supplementary Figures S1**-**S4**.

**Table 1:** Antibodies/streptavidin used in cytofluorimetry.

Specificity	Dilution	Host	Clone	Label	Producer
anti-CD4	1:80	Rat	GK1.5	eF450	Thermo Fisher Scientific
anti-CD8a	1:100	Rat	53-6.7	FITC	Thermo Fisher Scientific
anti-CD8a	1:80	Rat	53-6.7	PerCP-Cy5.5	Thermo Fisher Scientific
anti-CD25	1:160	Rat	PC61.5	PE	Thermo Fisher Scientific
anti-CD25	1:200	Rat	PC61.5	biotin	Thermo Fisher Scientific
anti-CD62L	1:80	Rat	MEL-14	PE-Cy7	Thermo Fisher Scientific
anti-CD62L	1:160	Rat	MEL-14	APC-eF780	Thermo Fisher Scientific
anti-Ki67	1:10	Rat	SolA15	FITC	Thermo Fisher Scientific
anti-IFN-γ	1:80	Rat	XMG1.2	PE	Thermo Fisher Scientific
anti-IL-17	1:80	Rat	TC11-18H10.1	APC	Biolegend
anti-IL-10	1:40	Rat	JES5-16E3	PerCP-Cy5.5	Thermo Fisher Scientific
anti-TNF	1:40	Rat	MP6-XT22	PE-Cy7	BD Biosciences
anti-Foxp3	1:20	Rat	FJK-16s	APC	Thermo Fisher Scientific
anti-CD11b	1:100	Rat	M1/70	FITC	Thermo Fisher Scientific
anti-CD11c	1:40	Armenian hamster	N418	PE-Cy5	Thermo Fisher Scientific
anti-MHC class II	1:300	Rat	M5/114.15.2	APC	Thermo Fisher Scientific
anti-B220	1:80	Rat	RA3-6B2	AF700	Thermo Fisher Scientific
anti-F4/80	1:40	Rat	BM8	BV510	Biolegend
anti-CD103	1:200	Hamster	O2E7	PE	Thermo Fisher Scientific
anti-CD40	1:40	Armenian hamster	HM40-3	eF450	Thermo Fisher Scientific
anti-PD-1	1:40	Armenian hamster	J43	FITC	Thermo Fisher Scientific
anti-CTLA-4	1:80	Armenian hamster	UC10-4B9	PE	Thermo Fisher Scientific
anti- KLRG1	1:80	Syrian hamster	2F1	PE-eF610	Thermo Fisher Scientific
anti-CD44	1:80	Rat	IM7	PerCP-Cy5.5	Thermo Fisher Scientific
anti-CD69	1:80	Armenian hamster	H1.2F3	PerCP	Biolegend
streptavidin	1:160			APC-Cy7	Biolegend

### 2.8 Statistical analysis

Statistical analysis was performed using GraphPad Prism 9 software (GraphPad Software, San Diego, CA). The significance of differences between groups was determined using a two-tailed Student’s t-test, Welch t-test or a one-way ANOVA test followed by a Tukey *post hoc* test, as indicated in the figure legends. Pearson’s correlation was used to calculate the correlation coefficient between cells. A p-value of less than 0.05 was considered statistically significant.

## 3 Results

### 3.1 CLP exerts transient effects on blood glucose regulation

**Figure 1:**
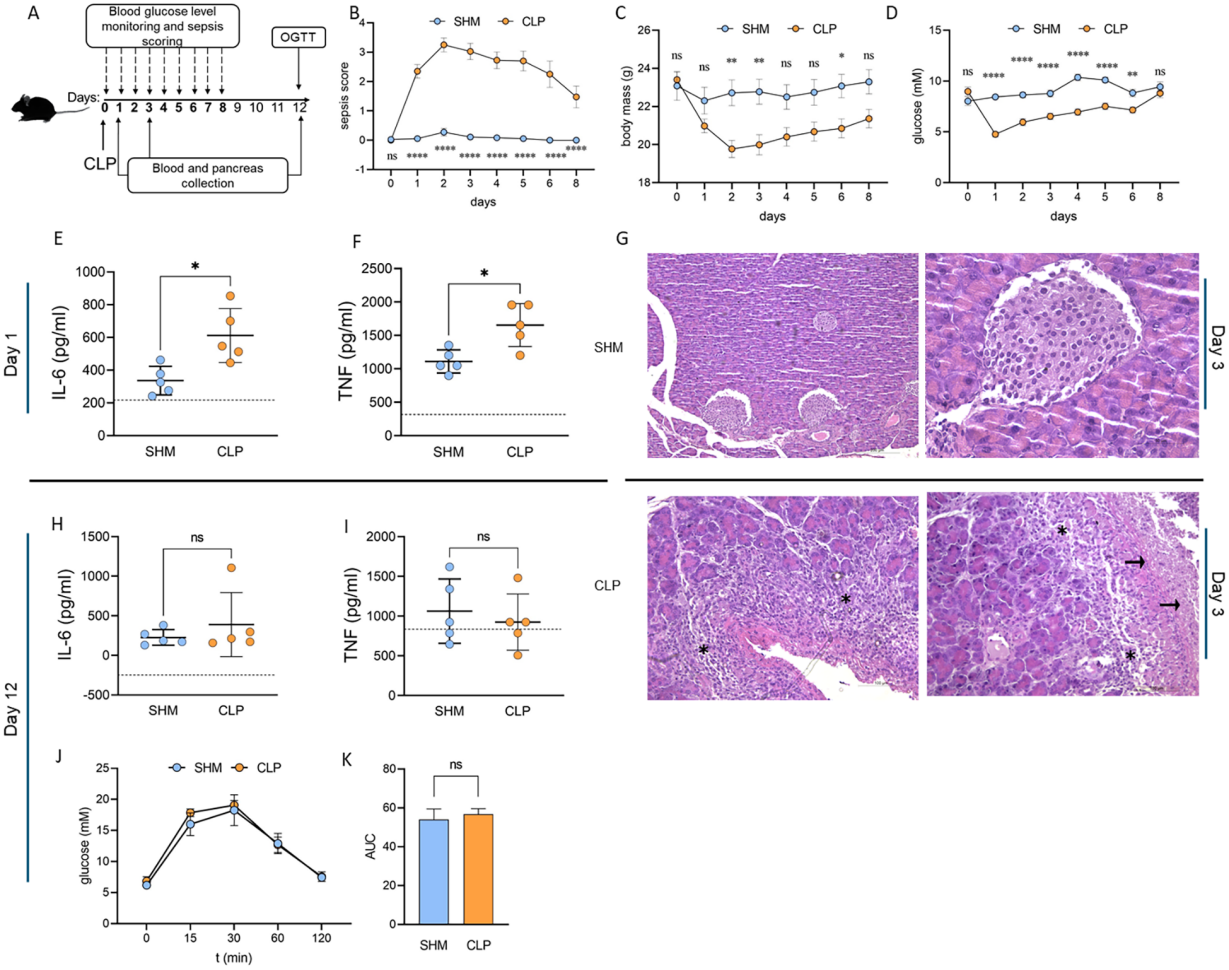
Effects of CLP-induced sepsis on blood glucose and cytokine levels. Experimental design **(A)**: C57BL/6 mice underwent either SHM (n= 18) or CLP (n= 20) surgery. In both groups, blood glucose level was monitored daily for 8 days post-surgery. On day 12, when all the animals from the CLP group showed no signs of sepsis, a glucose tolerance test was performed. Pancreases were obtained from animals of both groups on days 3 and 12. Clinical signs of sepsis in animals from SHM and CLP **(B)**. Body mass **(C)** and glucose levels **(D)** were monitored daily from day 0–8 post-surgery. Serum cytokine concentrations were measured 24h post-surgery and 12 days post-surgery for IL-6 **(E, H)** and TNF **(F, I)**. Representative histological sections **(G)** of the SHM (magnification x10 and x40) and CLP pancreas (magnification x20 and x40) at day 3 and 12 post-surgery showing acute inflammation (*) and necrosis (arrows) in CLP and healthy islets in SHM. Glucose levels **(J)** measured on day 12 during glucose tolerance test (n=6). AUC **(K)** derived from OGTT **(J)**. Data are presented as mean ± SEM (B, C, D, J), or mean ± SD **(E, F, H, I, K)**. ns, not significant. *P<0.05, **P<0.01, ***P<0.001, ****P<0.0001, by two-way ANOVA with Bonferroni’s multiple comparison test (B, C, D, J), Welch’s t-test **(E, F, H, I)** or Student’s t-test **(K)**.

To examine the influence of the immunoparalysis phase of sepsis on the development of the autoimmune process in the T1D model, C57BL/6 mice were exposed to polymicrobial sepsis induced by CLP. On day 12 after the CLP surgery, it was examined whether the cytokine storm had been resolved and function of the pancreas had been restored (**Figure 1A**). Sepsis was effectively induced in C57BL/6 mice by CLP, as evidenced by sepsis score and body mass loss (**Figures 1B, C**). To explore the extent to which CLP affected pancreatic function on its own, the blood glucose level was monitored daily from the day of surgery for 8 days, as indicated on the scheme (**Figure 1D**). In comparison to SHM mice, CLP mice had hypoglycemia, especially in the first few days (**Figure 1D**). However, by day 8, when symptoms of sepsis were declining, levels of blood glucose were back to values observed before CLP induction. In addition, levels of pro-inflammatory cytokines IL-6 and TNF that were significantly elevated in the first days after the CLP procedure (**Figures 1E, F**), returned to the levels seen in mice that underwent sham surgery (SHM) by day 12 post-surgery (**Figures 1H, I**). However, by day 8, when symptoms of sepsis were declining, levels of blood glucose were back to values observed before CLP induction. Further, acute inflammation and the necrosis of pancreatic acini were observed in CLP, but not in SHM mice, in the histology sections on day 3 post-surgery (**Figure 1G**). The glucose tolerance test, performed on day 12 when all the mice had completely recovered from the surgery, was performed to examine the pancreatic function, and it showed no difference between CLP and SHM mice (**Figures 1J, K**). Moreover, inflammation of the pancreas was not observed on histological sections of CLP mice at the same time (data not shown). These results show how sepsis transiently affects the pancreas and glucose regulation. Based on these data, in order to ensure that T1D is induced in mice having functional blood glucose regulation, MLDS induction of T1D in the following experiments started on day 12 after the surgery, *i.e.* at the time when the blood glucose levels recovered, and cytokine levels returned to basal values.

### 3.2 CLP prevents MLDS T1D

**Figure 2:**
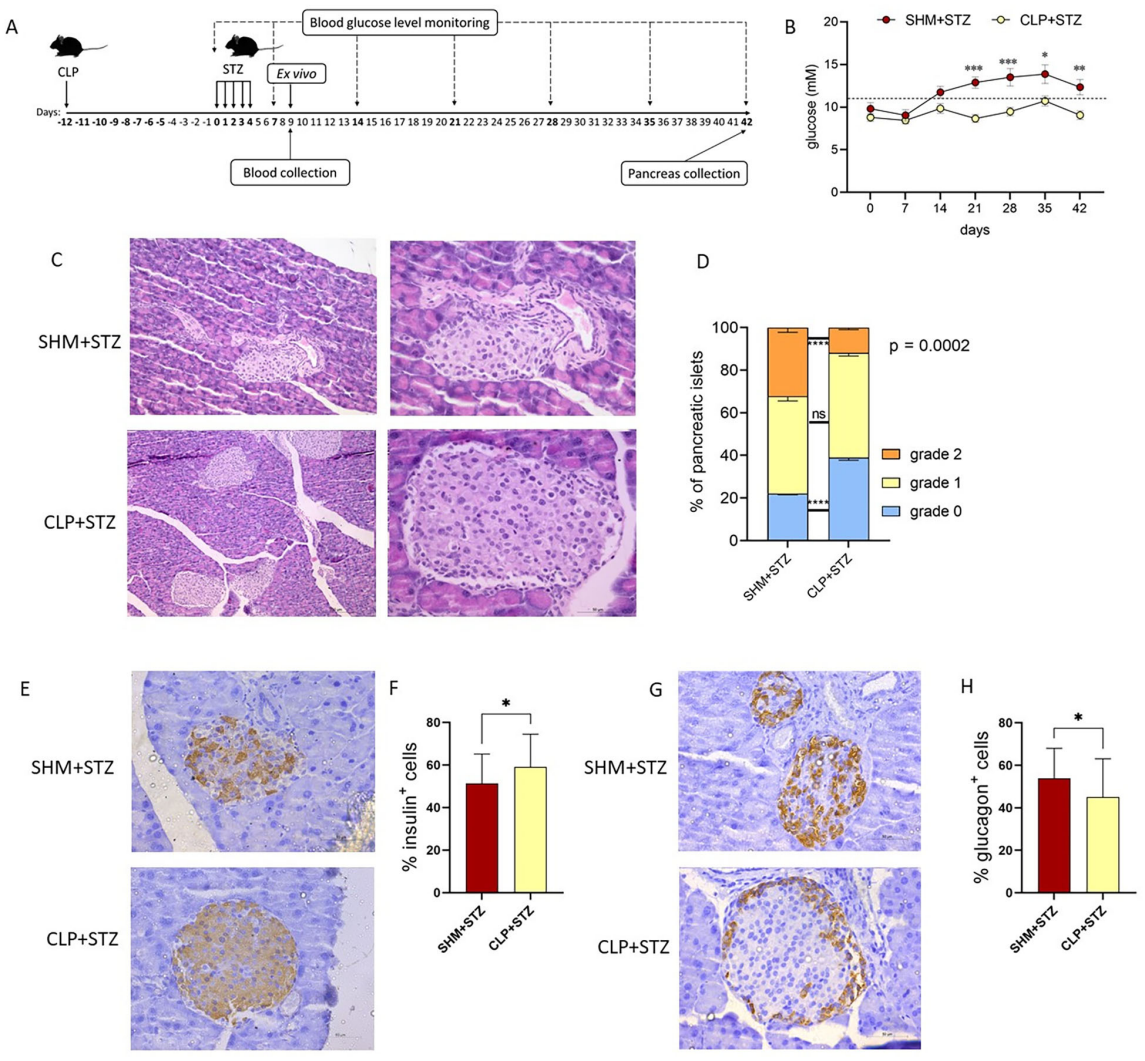
Diabetic parameters in MLDS T1D after sepsis. Experimental design **(A)**: animals underwent sham (n=18) or CLP (n=20) surgery. On day 12 after the surgery (day 0 of T1D), the T1D was induced in C57BL/6 mice by injecting low doses of streptozotocin (40 mg/kg BW) for 5 consecutive days. Blood glucose levels were monitored weekly, starting from the first day of STZ injection **(B)**. Dashed line represents the threshold for hyperglycemia. Representative histological sections of SHM+STZ and CLP+STZ (n=4 per group) pancreatic islets in MLDS-treated mice at the study endpoint (day 42; magnification x10 and x40 for SHM+STZ and magnification x20 and x40 for CLP+STZ) **(C)**. The percentages of healthy pancreatic islets (grade 0), islets with predominantly peri-ductal insulitis (grade 1) and peri-islet insulitis (grade 2) were calculated based on counting in the histological sections of pancreata (**D**, n = 4 per group). Representative histological sections of pancreatic islets from SHM+STZ and CLP+STZ animals stained for insulin **(E)** or glucagon **(G)** on day 42 (magnification x40). Percentage of insulin **(F)** and glucagon **(H)** positive cells in histological sections of SHM+STZ and CLP+STZ mice. Data are presented as mean ± SEM **(B)**, or mean ± SD **(D, F, H)**. ns, not significant. *P<0.05, **P<0.01, ***P<0.001, ****P<0.0001, by two-way ANOVA with Bonferroni’s multiple comparison test **(B)** or Student’s t test **(D, F, H)**.

To examine the effects of sepsis on T1D, the MLDS model was used to induce T1D in mice that were either sham-operated (SHM+STZ) or subjected to CLP (CLP+STZ) (**Figure 2A**). SHM+STZ mice developed hyperglycemia on day 14 after the first STZ injection and remained hyperglycemic until the end of the observation period (day 42). On the contrary, CLP+STZ mice were normoglycemic throughout the observation period (**Figure 2B**). Accordingly, histological assessment of pancreatic tissue in CLP+STZ animals showed dominantly preserved islets, in number, size and shape, or mild peri-ductal and peri-islet inflammation. At the same time, islets in SHM+STZ mice had slightly irregular contours and more pronounced peri-ductal and peri-islet inflammatory infiltrate (**Figures 2C, D**). Also, there was a higher percentage of insulin- and a lower percentage of glucagon-positive cells in the islets of the CLP+STZ group, compared to the islets of the SHM+STZ group (**Figures 2E-H**). Thus, these results suggest sepsis can induce resistance to STZ-induced T1D in mice.

### 3.3 CLP modulates PLN T cells in T1D

**Figure 3:**
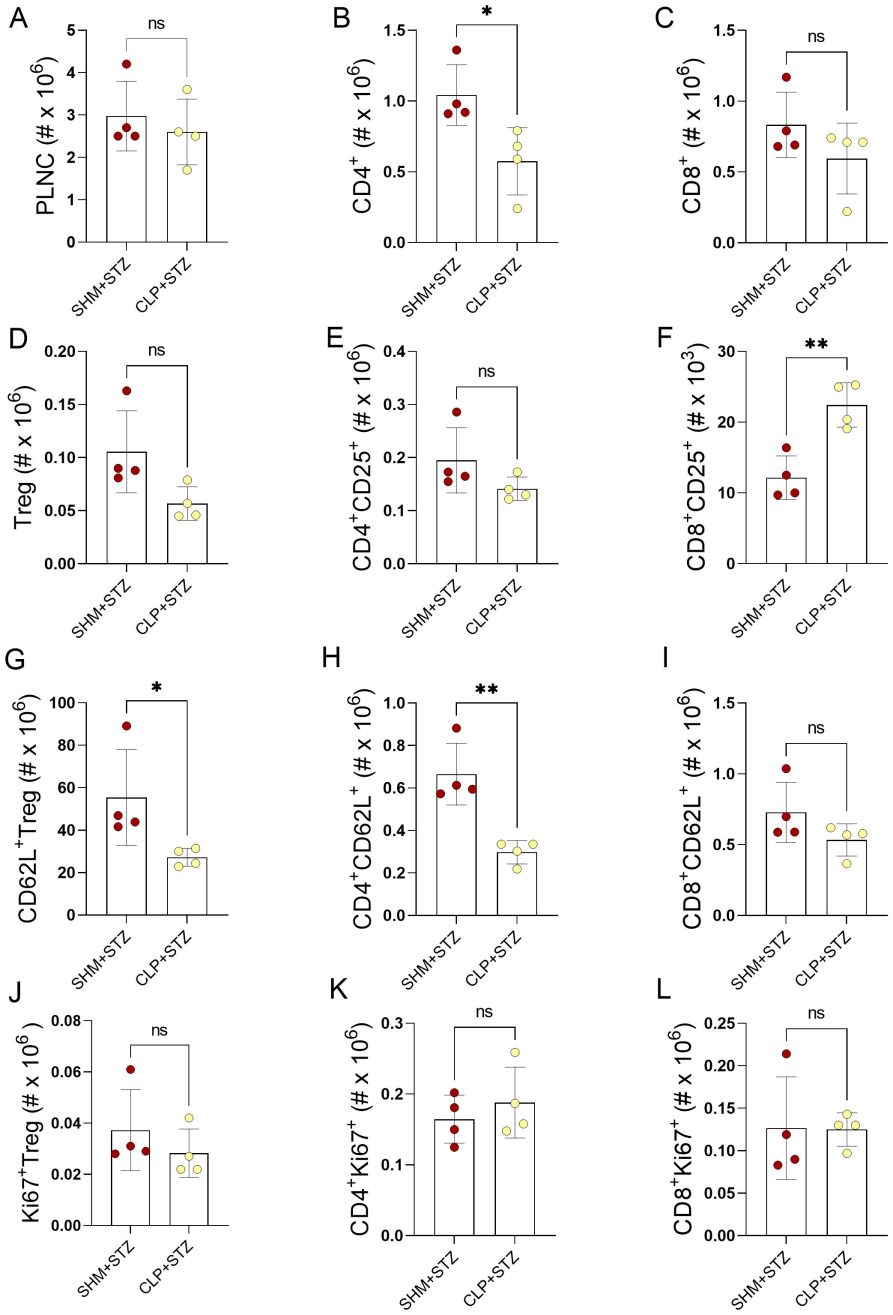
Phenotypical analysis of PLN T cells in MLDS T1D after sepsis. T1D was induced in SHM or CLP mice 12 days after the surgery. On day 9 after the first injection of STZ, immune cells were isolated from PLN and analyzed by flow cytometry. Absolute number of total cells in PLN **(A)**. Number of CD4^+^ cells **(B)** and CD8^+^ cells **(C)** in SHM+STZ and CLP+STZ mice. Number of Treg (CD4^+^CD25^+^FoxP3^+^) cells **(D)**. Number of CD4^+^CD25^+^ **(E)** and CD8^+^CD25^+^ cells **(F)**. Number of CD62L^+^ Treg **(G)**, CD4^+^CD62L^+^ cells **(H)**, and CD8^+^CD62L^+^ cells **(I)**. Number of Ki67^+^ Treg **(J)**, CD4^+^Ki67^+^ cells **(K)** and CD8^+^Ki67^+^ cells **(L)**. Data are presented as mean ± SD (n = 4 per group). ns, not significant. *P<0.05, **P<0.01, by Student’s t-test.

After we have observed that T1D clinical course was successfully ameliorated, we wanted to see how it affected immune cells in the PLN before examining the effect of the CLP on immune response related to T1D pathology. For this reason, we have isolated PLNC from SHM and SHM+STZ mice and examined main T cell populations involved in T1D, CD4^+^, CD8^+^ and Treg and their activation, proliferation and migratory marker. We did not observe any statistically significant changes induced by T1D, except for more proliferating CD4^+^ and Treg in SHM+STZ group (**Supplementary Figure S5**, **S6**). We next investigated the effects of sepsis on the cells of PLN, as the lymph nodes draining the pancreas, were investigated after STZ treatment. We were particularly interested in CD4^+^ T cells as the orchestrators of the immune attack on the β-cells, and CD8^+^ T cells as one of the major executors of β-cell destruction. Whereas the absolute cell number of PLN was without significant difference between SHM+STZ and CLP+STZ groups (**Figure 3A**), the number of CD4^+^ T cells and naïve (CD62L^+^) CD4^+^ T cells was decreased in CLP+STZ group (**Figures 3B, H**). The number of activated (CD25^+^) and proliferating (Ki67^+^) CD4^+^ T cells were similar in the examined groups (**Figures 3E, K**), as were the number of Treg (CD4^+^CD25^+^FoxP3^+^) and proliferating Treg (**Figures 3D, J**). We did find the number of CD62L^+^ Treg was decreased in the CLP+STZ group (**Figure 3G**). As for CD8^+^ T cells, there was no change in the number of all, CD62L^+^, and proliferating cells (Ki67^+^) (**Figures 3C, I, L**), but there were more CD25^+^ CD8^+^ T cells in the CLP+STZ group (**Figure 3F**). Thus, CLP influenced the T cell profile in the PLN of MLDS-treated mice.

**Figure 4:**
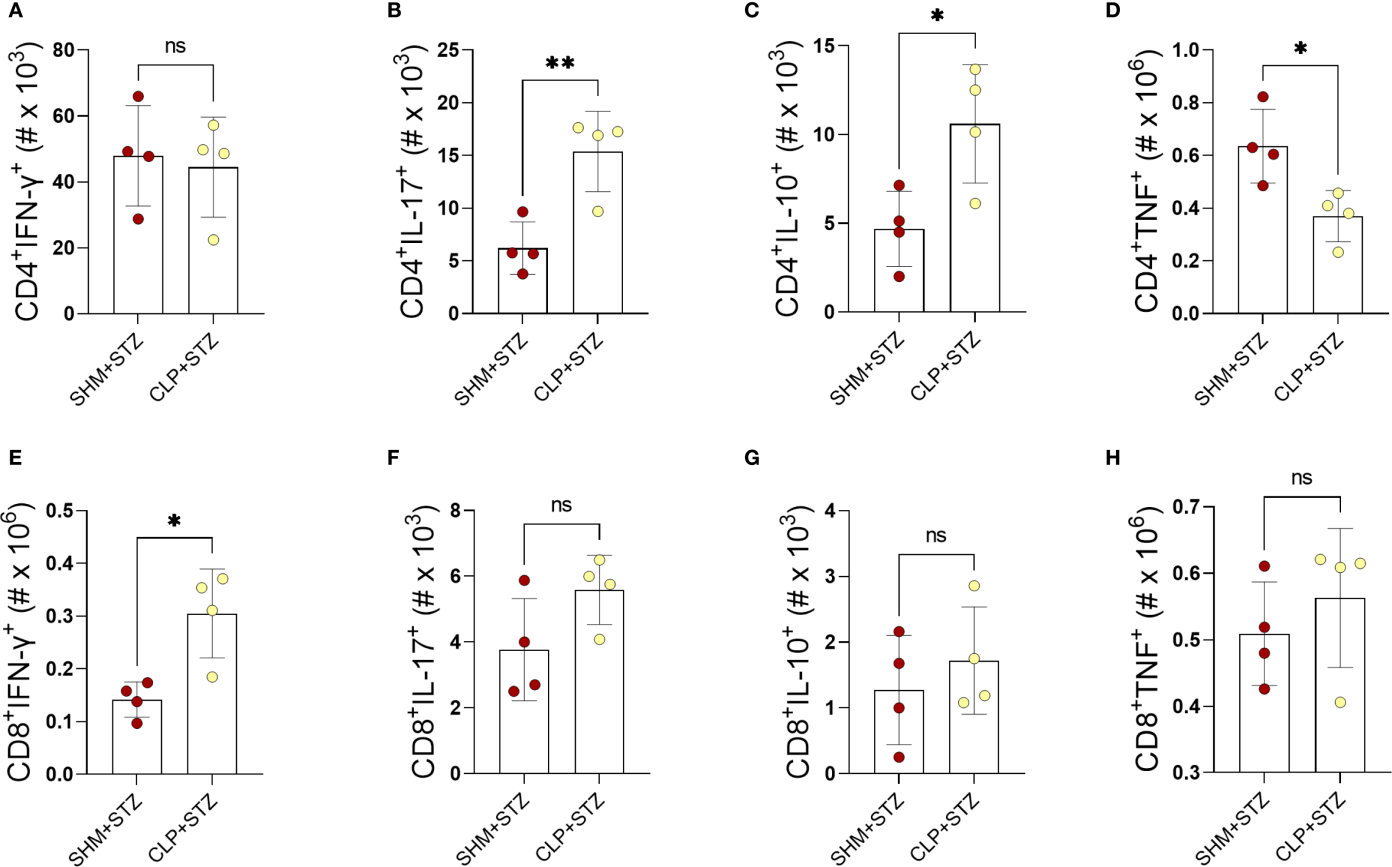
The number of cytokine-producing PLN T cells in MLDS T1D after sepsis. The number of CD4^+^ **(A-D)** and CD8^+^ **(E-H)** T cells producing IFN-γ **(A, E)**, IL-17 **(B, F)**, IL-10 **(C, G)**, and TNF **(D, H)**, was determined by flow cytometry in cells isolated from the PLN on day 9 after the start of the T1D induction. Data are presented as mean ± SD (n = 4 per group). ns, not significant. *P<0.05, **P<0.01, by Student’s t-test.

To further delineate the effects of sepsis on PLN T cells in MLDS T1D, we determined the number of CD4^+^ and CD8^+^ T cells able to produce IFN-γ, IL-17, TNF and IL-10 in CLP+STZ and SHM+STZ mice. A significant increase in the number of IL-17- and IL-10-producing, and a decrease of TNF-producing CD4^+^ T cells was observed in CLP+STZ mice, as well as an increase in the number of IL-17- and IFN-γ-producing CD8^+^ T cells (**Figures 4B-E**). The other examined populations did not differ significantly between the groups (**Figures 4A, F-H**). Thus, sepsis also altered the cytokine-producing capacity of PLN T cells in T1D mice.

### 3.4 CLP has a limited effect on antigen-presenting cells in MLDS T1D

**Figure 5:**
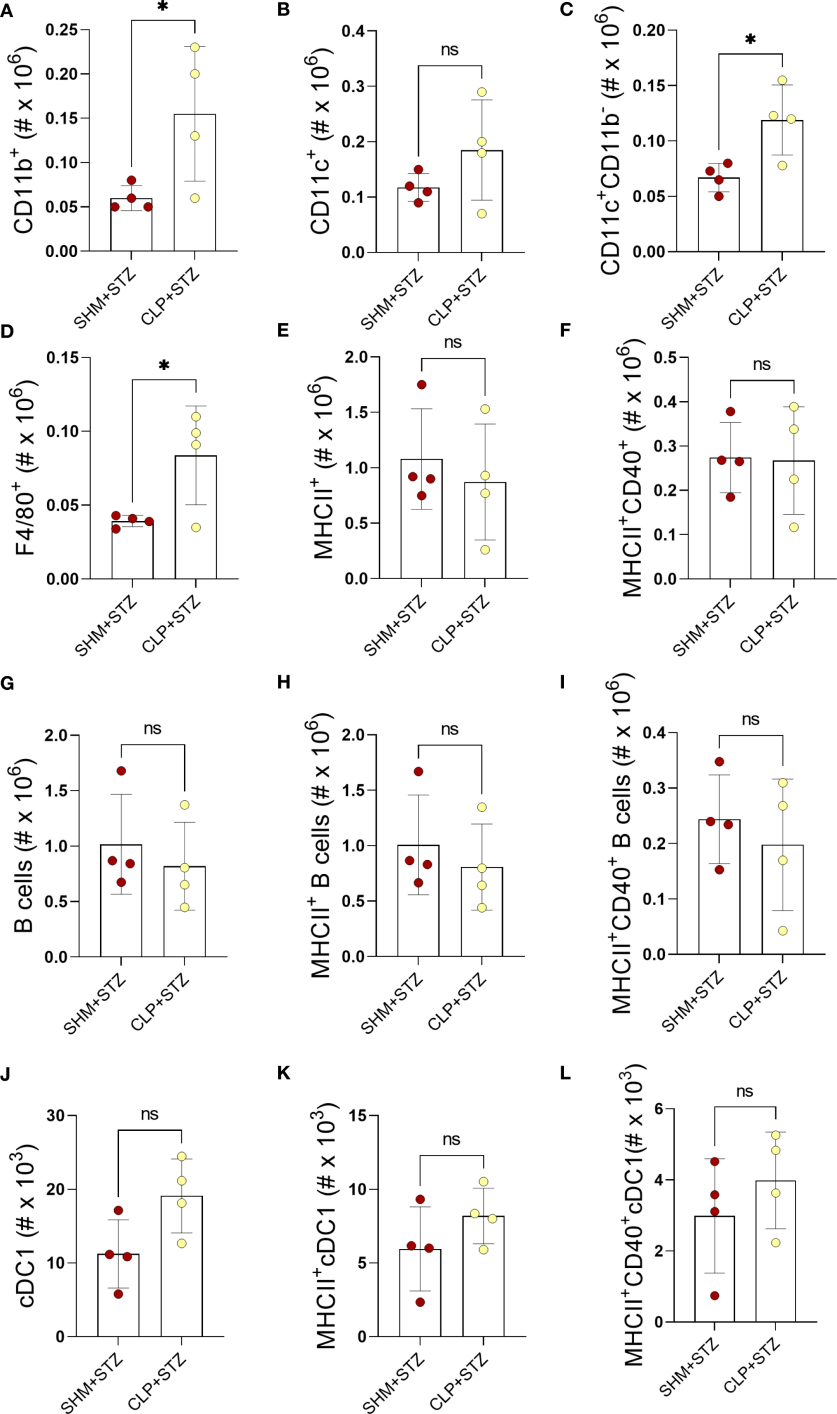
The number of PLN antigen-presenting cells in MLDS T1D after sepsis. The number of CD11b^+^ cells **(A)**, CD11c^+^ cells **(B)**, CD11c^+^CD11b^-^ **(C)**, F4/80^+^ cells **(D)**, MHCII^+^ cells **(E)**, MHCII^+^CD40^+^ cells **(F)**, B cells (B220^+^) **(G)**, MHCII^+^ B cells **(H)**, CD40^+^MHCII^+^ B cells **(I)**, cDC1 (CD11c^+^CD11b^-^CD103^+^ cells) **(J)**, MHCII^+^ cDC1 **(K)**, MHCII^+^CD40^+^ cDC1 **(L)** in PLN was determined by flow cytometry on day 9 after the first STZ injection. Data are presented as mean ± SD (n = 6 per group). ns, not significant. *P<0.05, by Student’s t-test.

To further our interrogation of sepsis on the generation of T1D, we determined the impact of sepsis on antigen-presenting PLN populations in MLDS T1D. First, we aimed to exclude the effect of T1D by comparing SHM+STZ with their control, SHM, and observed no differences in APC populations between these groups (**Supplementary Figure S7**). Next, we focused on the changes induced by sepsis in T1D. While CLP increased the number of CD11b^+^, CD11c^+^CD11b^-^ cells, and F4/80^+^ macrophages (**Figure 5A, C, D**), it did not affect the number of CD11c^+^ (**Figure 5B**) nor examined populations of MHC class II-positive cells, B cells and a subset of conventional dendritic cells (cDC1), CD11c^+^CD11b^-^CD103^+^ DC (**Figures 5E-L**). Thus, a limited influence of sepsis on the abundance of PLN antigen-presenting cells was observed in MLDS-treated mice.

### 3.5 CLP modulates CD4+ T cells in the spleen

**Figure 6:**
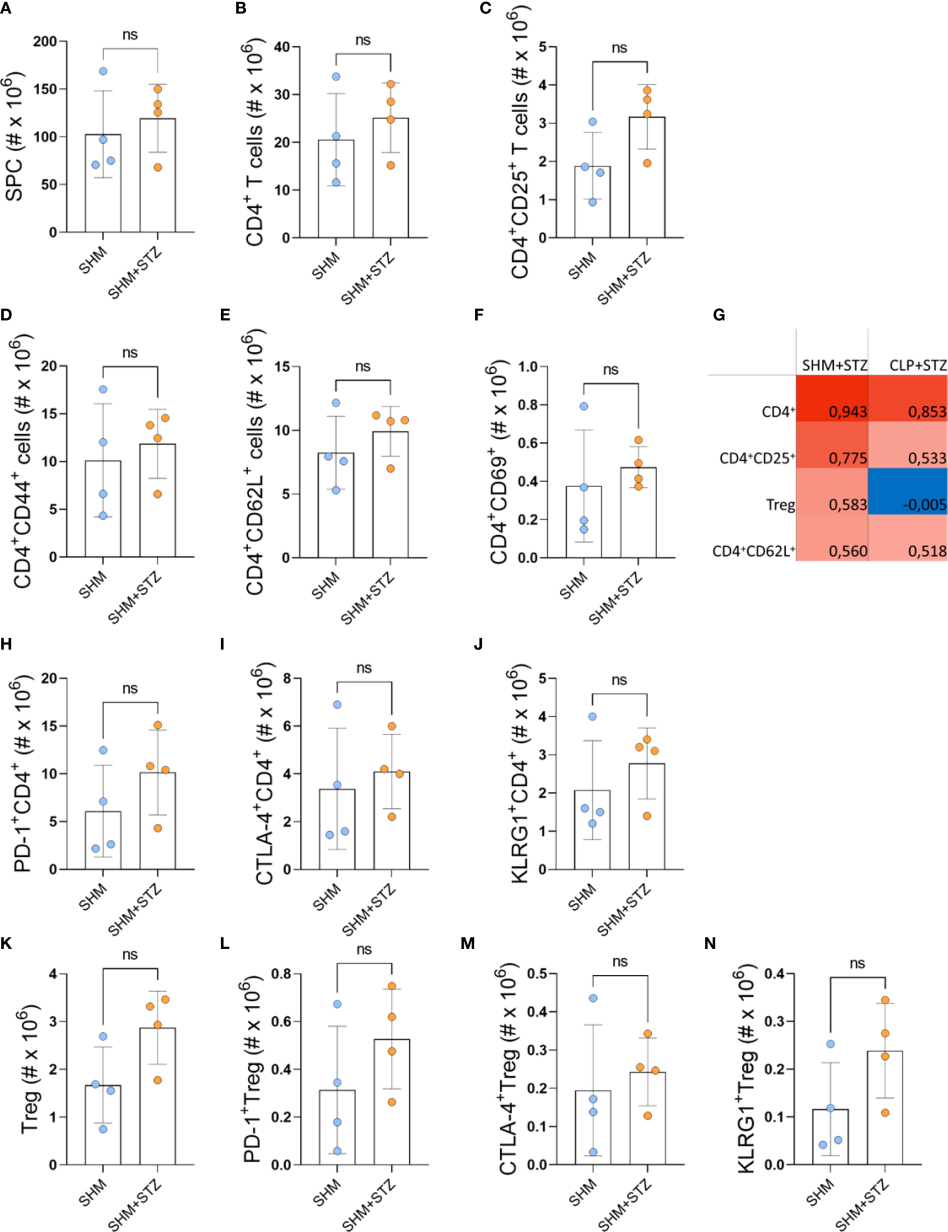
The influence of sepsis on the activation and exhaustion profile of T cells in MLDS T1D. The absolute number of SPC isolated on day 9 after the induction of T1D **(A)**. The number of CD4^+^ cells **(B)**, CD4^+^CD25^+^ cells **(C)**, CD4^+^CD44^+^ cells **(D)**, CD4^+^CD62L^+^ cells **(E)**, CD4^+^CD69^+^ cells **(F)**. The number of PD-1^+^CD4^+^ cells **(H)**, CTLA-4^+^CD4^+^ cells **(I)**, KLRG1^+^CD4^+^ cells **(J)**, Treg (CD4^+^CD25^+^FoxP3^+^) **(K)**, PD-1^+^ Treg **(L)**, CTLA-4^+^ Treg **(M)** and KLRG1^+^ Treg **(N)**. Data are presented as mean ± SD (n = 4 per group). ns, not significant. *P<0.05, **P<0.01, ***P<0.001, by Student’s t-test. Heatmap showing Pearson’s correlation coefficient of different cell populations (CD4^+^, CD4^+^CD25^+^, Treg, CD4^+^CD62L^+^) calculated between the PLNC and SPC in SHM+STZ and CLP+STZ mice **(G)**.

To assess systemic effects of mice pre-exposure to sepsis on CD4^+^ T cells, SPC were analyzed in parallel with PLN. T1D did not cause any statistically significant changes in the spleen (**Supplementary Figure S8**), unlike sepsis, which caused an increase in the absolute number of SPC in control animals that were not subjected to T1D (**Supplementary Figure S9A**). After induction of T1D, there was no difference in the number of CD4^+^, CD4^+^CD25^+^, CD4^+^CD44^+^ T cells, and Treg in the spleens of CLP+STZ and SHM+STZ mice (**Figures 6A-D, K**). However, a significant decrease of CD62L^+^ and an increase of CD69^+^ CD4^+^ T cells were observed in CLP+STZ splenocytes (**Figures 6E, F**). There was no difference in CD4^+^, CD4^+^CD25^+^, CD4^+^CD62L^+^, CD4^+^CD44^+^, CD4^+^CD69^+^ T cells, and Treg in the spleens of SHM and CLP group (**Supplementary Figure S9B-F, J**). To explore whether the immune changes in the draining PLN are coordinated with the systemic immune changes, we performed correlation analysis between PLNC and SPC within each experimental group after the T1D induction (**Figure 6G**). Positive correlation of similar extent was observed for CD4^+^, CD4^+^CD25^+^, and CD4^+^CD62L^+^ cells in SHM+STZ and CLP+STZ mice. However, while a positive correlation for Treg was observed in the SHM+STZ group, there was a lack of correlation in the CLP+STZ group. This suggests that pre-exposure to sepsis alters the local/systemic distribution of Tregs in T1D.

Expression of inhibitory/exhaustion markers (PD-1, CTLA-4, KLRG1) in CD4^+^ T cells was explored next. The number of cells expressing each of the examined markers was significantly higher in the CLP+STZ group (**Figures 6H-J**). This effect was also observed when comparing SHM and CLP groups (**Supplementary Figure S9G-I**). Although the number of Treg was not altered after sepsis (**Supplementary Figure S9J**, **Figure 6K**), a higher number of Treg expressing inhibitory markers was noticed, specifically a higher number of KLRG1^+^ Treg in CLP compared to SHM animals (**Supplementary Figure S9M**), and a higher number of PD-1^+^ and CTLA-4^+^, but not of KLRG1^+^ Treg in CLP+STZ compared to SHM+STZ (**Figures 6L-N**). Thus, pre-exposure of mice to sepsis led to the change of phenotypic/functional properties of CD4^+^ T cells, and Treg in particular, in the spleen.

## 4 Discussion

Although infection is generally considered a contributing factor in T1D pathogenesis, reports are showing that infection with specific bacteria or helminths can be protective in T1D (24, 25). Specifically, infection with *Mycobacterium avium, Schistosoma mansoni, Salmonella typhimurium, Heligmosomoides polygyrus* (26–29) prevented the development of T1D in NOD mice. These results are in agreement with our own, showing that septic events reduce susceptibility of mice to the induction of T1D. The effect of sepsis on the induction of T1D in our study is associated with the altered activation profile of T cells, CD4^+^ T cells in particular, in the pancreas-related lymphoid organs. It is also important to note that the number of CD4^+^ T cells and Treg expressing exhaustion/regulation markers was increased in the spleen of mice that first experienced a septic event.

The lower number of CD4^+^ T cells present in the PLN of CLP-treated mice is in agreement with previously published data showing a decrease in the total number of circulating lymphocytes, and CD4^+^ and CD8^+^ T-cells in particular, after CLP (30, 31). We also found that the number of CD62L-expressing CD4^+^ T cells and Treg in the PLN and spleen of CLP-treated mice was lower. CD62L^+^ T cells are considered naïve or central memory cells that home to secondary lymphoid tissue, where they expand and provide helper functions. In a study by Aljobaily et al. (32), it was demonstrated that a continuous influx of stem-like CD62L^+^ T cells is necessary to sustain autoimmunity. By depleting or blocking their migration T cell number in the islets is reduced, and the progression of the disease is delayed. This is in accordance with our results, where we observed a lower number of CD62L^+^ T cells in the CLP+STZ group, which hadn’t developed hyperglycemia, compared to the SHM+STZ group, which had significantly higher numbers of these cells in both the PLN and spleen. Further, a higher number of IL-10-producing and lower number of TNF-producing CD4^+^ T cells in PLN substantiate the idea that immunoregulatory surroundings prevail in lymphoid organs after sepsis. Along the same line, the number of CD4^+^ T cells expressing important regulatory/exhaustion molecules (i.e., PD-1, CTLA-4, and KLRG1) was increased in the spleen of CLP-treated mice. The marked increase in PD-1 expression on T cells is consistent with a previous study indicating a higher mRNA level of PD-1 in the liver after CLP (33). In humans, sepsis also leads to an upregulation of PD-1 and CTLA-4 on T cells compared to healthy controls (34). Thus, the observed increased number of T cells expressing these markers implies that T cell exhaustion may play a role in the prevention of T1D development after sepsis. This is in agreement with the literature, where lower expression of PD-1 on CD4^+^ T cells was thought to contribute to the development and/or maintenance of T1D (35). Similar conclusions were obtained from the studies of CTLA-4 knock-out mice. Namely, these animals showed high accumulation of activated and highly proliferative lymphocytes in several tissues and organs, thus implying that CTLA-4 acts as a negative regulator of T cells. (36) However, the higher expression of these markers on Treg of CLP+STZ mice can be interpreted not in the context of immune suppression, but rather as their increased regulatory function. There is evidence showing that the lower expression of PD-1 on Treg is associated with their defective activation in T1D patients (35). Along the same line, it was shown that Treg can suppress immune response not only by soluble factors, like IL-10, but with cell-cell mediated mechanisms, through CTLA-4. As for KLRG1, studies are showing that CD8+ T cells isolated from T1D patients who were receiving treatment exhibited an exhausted phenotype and had higher expression of KLRG1 (37). It could be speculated that a similar profile is seen in our results, where CD4^+^ T cells isolated from the PLN of CLP+STZ mice have higher expression of KLRG1. KLRG1 expression is shown to delineate a subpopulation of dysfunctional Treg cells during T1D progression in a mouse model (38). In our results, there are no differences between SHM+STZ and CLP+STZ animals in KLRG1^+^ Treg, thus supporting the idea that in CLP+STZ animals, CD4^+^ T cells exhibited an exhausted profile, while Treg maintained their functional capacity, providing beneficial effects in T1D model.

Interestingly, a higher number of IL-17-producing CD4^+^ T cells was observed in the PLN of CLP-treated mice. Although IL-17-producing T cells are generally considered pro-inflammatory and pathogenic in T1D (19, 39, 40), some data imply these cells can have the opposite activity. For instance, elevated numbers of Th17 cells in lymph nodes draining the site of a burn wound initiate and control post-burn immunosuppression (41). IL-17 can also promote maturation and function of Treg (42), and Th17 cells can acquire Treg functions (43). Importantly, in a study exploring the influence of segmented filamentous bacteria on T1D in NOD mice, Th17 cells were shown protective (44). The protective role of Th17 in T1D was corroborated with several other studies in NOD mice (45–47). Thus, the accumulation of Th17 cells in T1D PLN after sepsis may reflect a response to commensal bacteria exposed to the immune system. Moreover, in light of the shown protective role of Th17 cells in NOD mice, we can assume that similar protective activity of Th17 cells is effective in our experimental setting. Thus, it will be necessary to explore the possibility that Th17 cells contribute to the regulatory environment in pancreas-associated lymphoid tissues in T1D.

We found it interesting that there was only a limited effect of sepsis pre-exposure on antigen-presenting cells, *i.e.* statistically significant increase was observed in CD11b^+^, CD11c^+^CD11b^-^ and F4/80^+^ cells, but not in CD11c^+^CD11b^-^CD103^+^ DC in the CLP+STZ group. Importantly, there was no increase in the number of MHCII^+^ or MHCII^+^CD40^+^ cells among the investigated populations. Thus, the increased total number of macrophages/DC do not transfer to their greater ability to perform antigen-presenting functions. Accordingly, there was no change in number of activated CD4^+^ T cells in PLN. Still, there was an increase in the number of activated CD8^+^ T cells, as well as of IFN-γ-producing CD8^+^ T cells after pre-exposure to sepsis. Although IFN-γ is generally considered essential for cytotoxic functions of CD8^+^ T cells (48), there are reports showing that IFN-γ negatively influences CD8^+^ T cells and controls expansion of CD8^+^ T cells, even in the context of autoimmunity (49–51). Hence, the observed increase in IFN-γ-producing CD8^+^ T cells might contribute to the limitation of T1D development in mice.

It will be important to test if the phenomenon of T1D prevention observed in the MLDS model is viable in other animal models, especially in NOD mice. The major advantage of NOD mice over MLDS is the ability to explore beta cell antigen-specific T cell response. Still, the major disadvantage for the use of NOD mice in our study is the inability to predict the exact onset of T1D in these mice, and thus to adjust the timing of CLP. Generally, NOD model is considered superior to MLDS regarding similarity with human T1D pathogenesis, yet MLDS model is also a reliable tool to study autoimmunity directed against the pancreatic β-cells (52). Further, the effect of sepsis pre-exposure on pancreatic immune cells was not examined in this study, and this analysis would provide valuable information on the phenomenon. Thus, further studies on the interaction of sepsis and T1D are perceivable. Taken together, our results suggest that sepsis may promote a state of immune exhaustion or tolerance in CD4^+^ T cells, helping to suppress the autoimmune attack on pancreatic β-cells. Further studies are needed to investigate this concept, as it suggests that sepsis reprograms the immune system to prevent or slow down the destruction of β-cells, a central feature of T1D pathogenesis. Even though this study lacks specific insights into potential mechanisms behind the observed effects, our data does indicate that some of the features of the immunoparalysis phase of sepsis, such as exhaustion of T cells and potentiation of Treg functions, could be employed to design novel pharmacological approaches to achieve dampening, or even prevention, of autoimmunity.

## Data Availability

The raw data supporting the conclusions of this article will be made available by the authors, without undue reservation.
