# Supplementary material for: Sepsis prevents the development of experimental type 1 diabetes
Source: Front Immunol. 2025 Oct 9;16:1658960. doi: 10.3389/fimmu.2025.1658960 (PMC12546358; doi:10.3389/fimmu.2025.1658960)
Supplement: Supplementary file 1 [file DataSheet1.pdf]

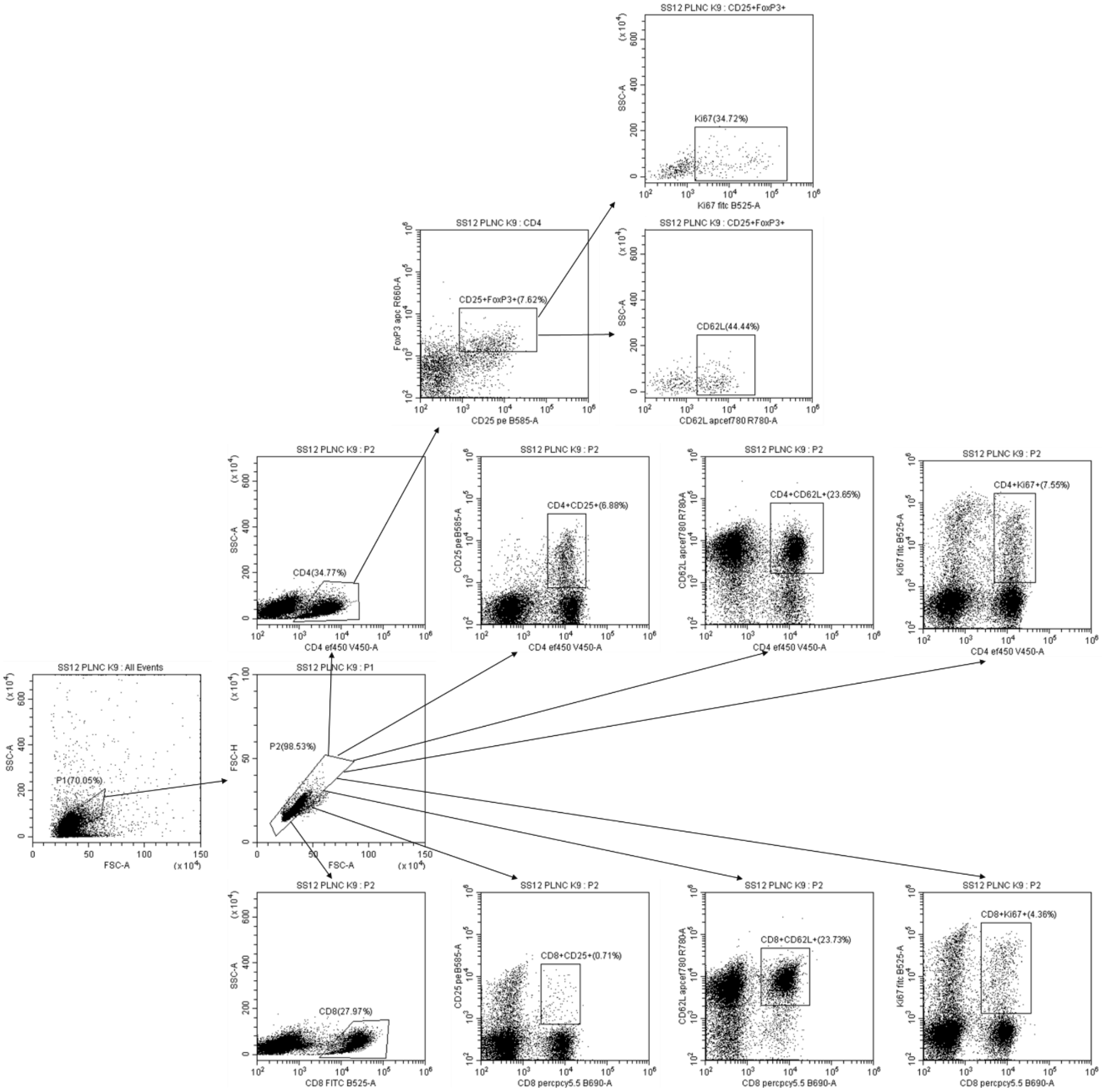

Figure S1. Representative flow cytometry plots showing gating strategy for the results presented in the Figure 3.

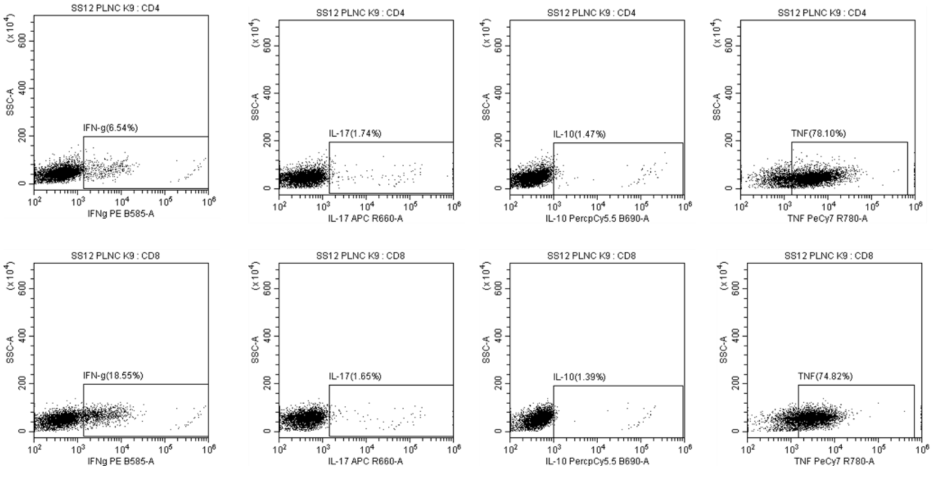

Figure S2. Representative flow cytometry plots showing gating strategy for the results presented in the Figure 4.

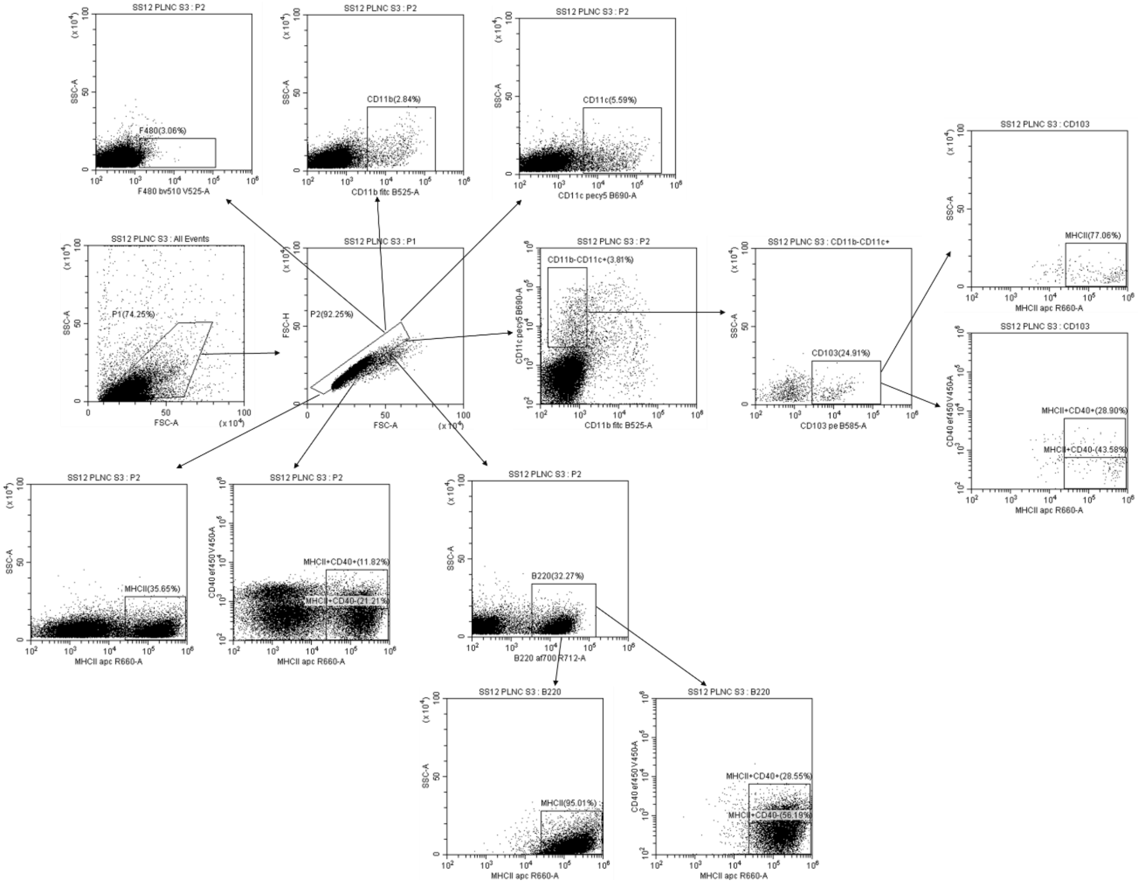

Figure S3. Representative flow cytometry plots showing gating strategy for the results presented in the Figure 5.

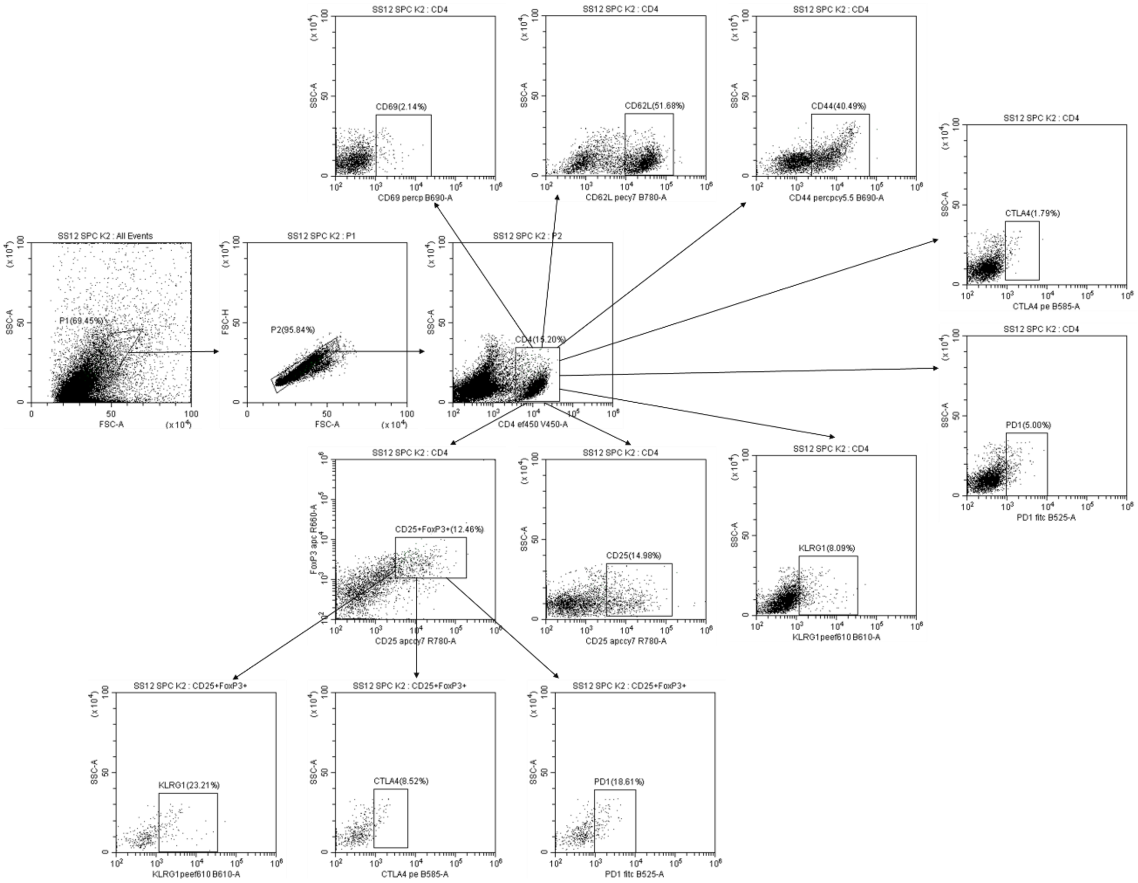

Figure S4. Representative flow cytometry plots showing gating strategy for the results presented in the Figure 6.

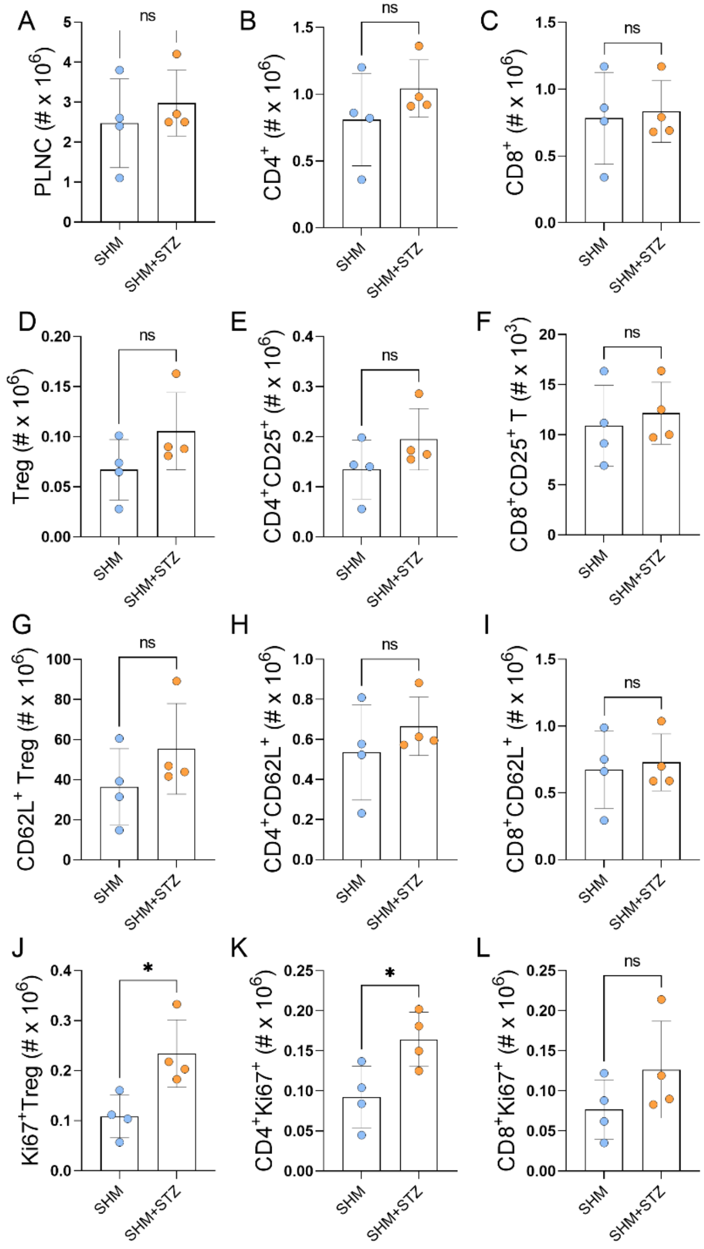

Figure S5. Phenotypical analysis of PLN T cells in MLDS T1D. T1D was induced in SHM mice by injecting 38mg/ml STZ in five consecutive days. On day 9 after the first injection of STZ, immune cells were isolated from PLN and analyzed by flow cytometry. Absolute number of total cells in PLN (A). Number of CD4$^+$ cells (B) and CD8$^+$ cells (C) in SHM+STZ and CLP+STZ mice Number of Treg (CD4$^+$CD25$^+$FoxP3$^+$) cells (D). Number of CD4$^+$CD25$^+$ (E) and CD8$^+$CD25$^+$ cells (F). Number of CD62L$^+$ Treg (G), CD4$^+$CD62L$^+$ cells (H), and CD8$^+$CD62L$^+$ cells (I). Absolute number of CD62L$^+$ Treg (J), CD4$^+$Ki67$^+$ cells (K) and CD8$^+$Ki67$^+$ cells (L). Data are presented as mean ± SD (n = 4 per group). ns, not significant. *P<0.05, **P<0.01, by Student's t-test.

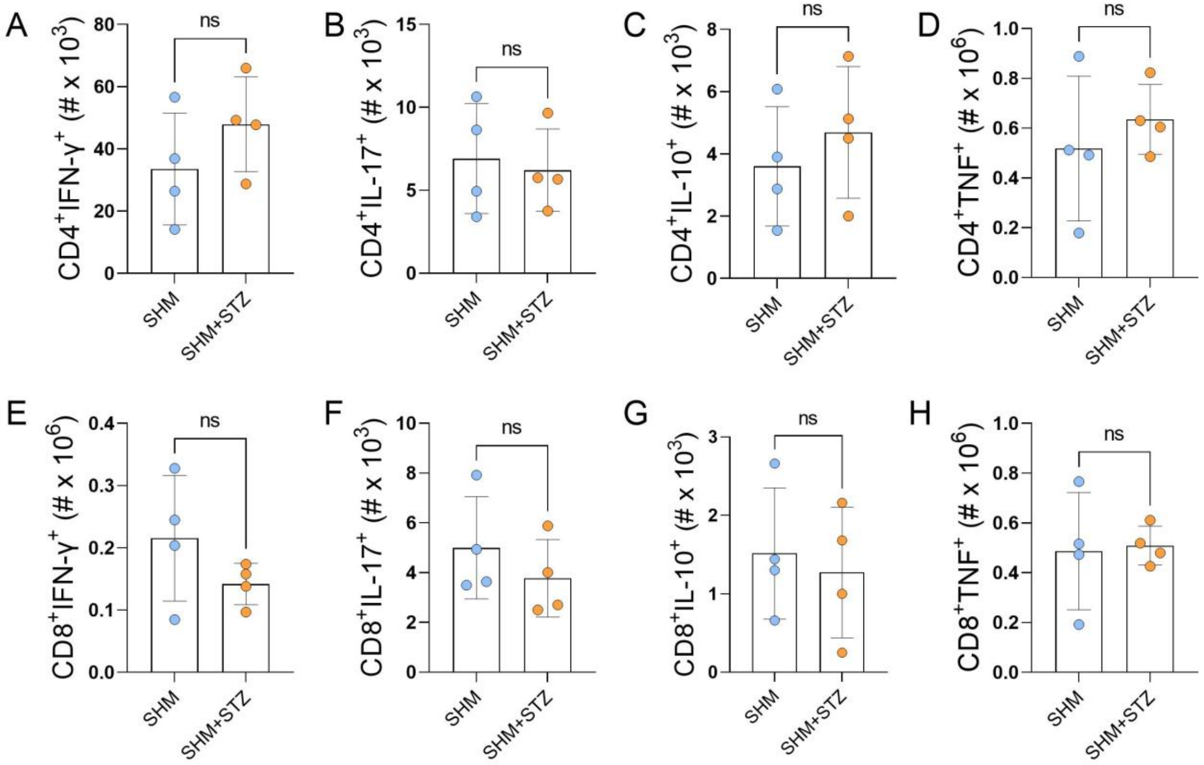

Figure S6. The number of cytokine-producing PLN T cells in MLDS T1D. The number of $CD4^+$ (A-D) and $CD8^+$ (E-H) T cells producing IFN-γ (A, E), IL-17 (B, F), IL-10 (C, G), and TNF (D, H), was determined by flow cytometry in cells isolated from the PLN on day 9 after the start of the T1D induction from SHM+STZ or control, SHM group. Data are presented as mean ± SD (n = 4 per group). ns, not significant. *P<0.05, **P<0.01, by Student's t-test.

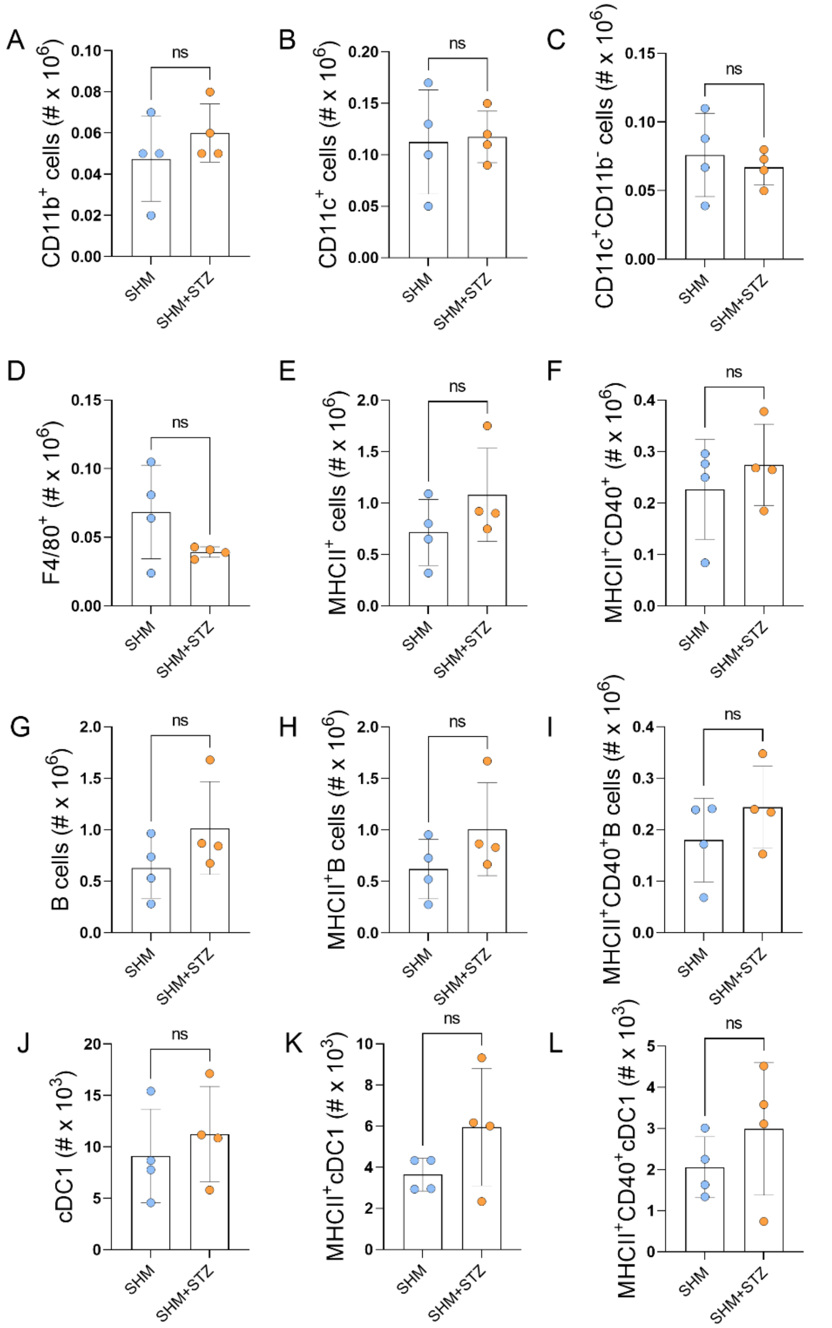

Figure S7. The number of PLN antigen-presenting cells in MLDS T1D. The number of CD11b$^+$ cells (A), CD11c$^+$ cells (B), CD11c$^+$CD11b$^-$ (C), F4/80$^+$ cells (D), MHCII$^+$ cells (E), MHCII$^+$CD40$^+$ cells (F), B cells (B220$^+$) (G), MHCII$^+$ B cells (H), CD40$^+$MHCII$^+$ B cells (I), cDC1 (CD11c$^+$CD11b$^-$CD103$^+$ cells) (J), MHCII$^+$ cDC1 (K), MHCII$^+$CD40$^+$ cDC1 (L) in PLN was determined by flow cytometry on day 9 after the first STZ injection. Data are presented as mean ± SD (n = 6 per group). ns, not significant. *P<0.05, by Student's t-test.

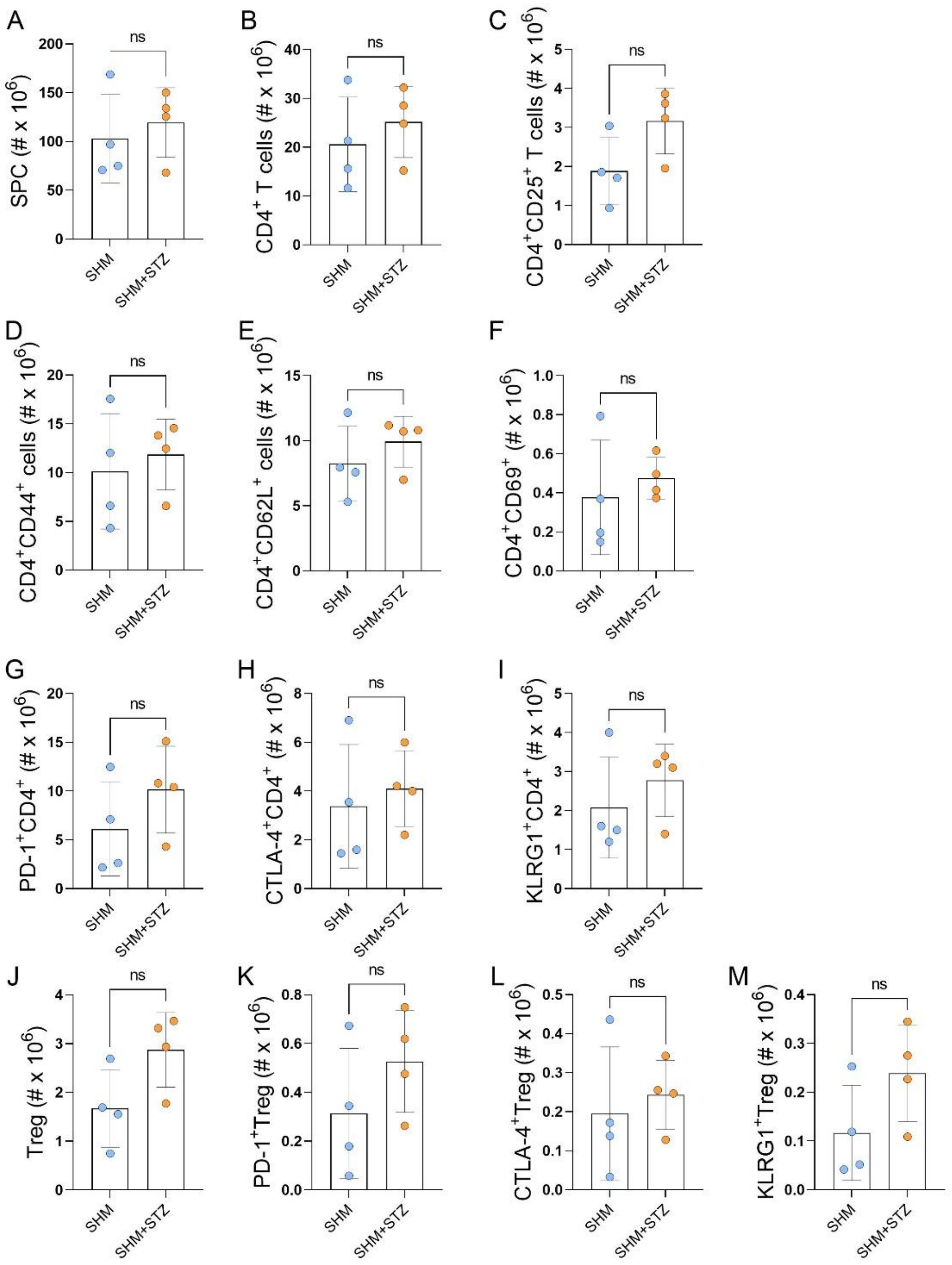

Figure S8. Activation and exhaustion profile of spleen T cells in MLDS T1D. The absolute number of SPC isolated on day 9 after the induction of T1D was measured by flow cytometry (A). The number of $CD4^+$ cells (B), $CD4^+CD25^+$ cells (C), $CD4^+CD44^+$ cells (D), $CD4^+CD62L^+$ cells (E), $CD4^+CD69^+$ cells (F). The number of PD-1^+CD4^+ cells (G), CTLA-4^+CD4^+ cells (H), $KLRG1^+CD4^+$ cells (I), Treg ($CD4^+CD25^+FoxP3^+$) (J), PD-1^+ Treg (K), CTLA-4^+ Treg (L) and $KLRG1^+$ Treg (M). Data are presented as mean ± SD (n = 4 per group). ns, not significant. *P<0.05, **P<0.01, ***P<0.001, by Student's t-test.

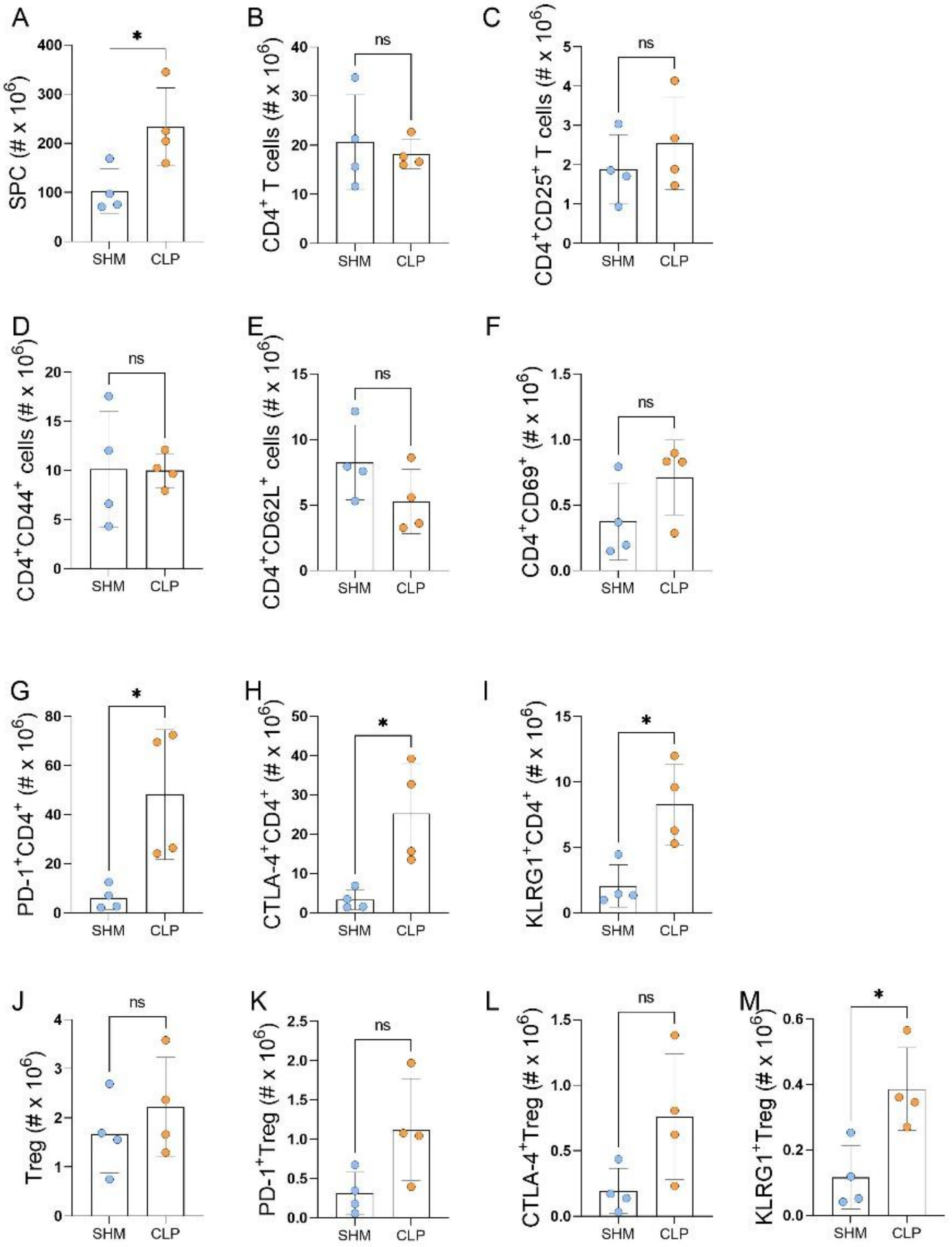

Figure S9. The influence of sepsis on T cell activation and exhaustion profile in the spleen. The absolute number of SPC isolated on day 9 after the induction of T1D was measured by flow cytometry (A). The number of CD4$^+$ cells (B), CD4$^+$CD25$^+$ cells (C), CD4$^+$CD44$^+$ cells (D), CD4$^+$CD62L$^+$ cells (E), CD4$^+$CD69$^+$ cells (F). The number of PD-1$^+$CD4$^+$ cells (G), CTLA-4$^+$CD4$^+$ cells (H), KLRG1$^+$CD4$^+$ cells (I), Treg (CD4$^+$CD25$^+$FoxP3$^+$) (J), PD-1$^+$ Treg (K), CTLA-4$^+$ Treg (L) and KLRG1$^+$ Treg (M). Data are presented as mean ± SD (n = 4 per group). ns, not significant. *P<0.05, **P<0.01, ***P<0.001, by Student's t-test.
